# Impact of micro- and nanoplastics exposure on human health: focus on neurological effects from ingestion

**DOI:** 10.3389/fpubh.2025.1681776

**Published:** 2025-10-31

**Authors:** Sudeepa Bhattacharyya, Melody L. Greer, Maryam Salehi

**Affiliations:** ^1^Department of Biomedical and Health Informatics, University of Missouri-Kansas City, Kansas City, MO, United States; ^2^Department of Biomedical Informatics, University of Arkansas for Medical Sciences, Little Rock, AR, United States; ^3^Department of Civil and Environmental Engineering, University of Missouri, Columbia, MO, United States

**Keywords:** microplastics, nanoplastics, environmental health, neurological effects, public health, plastic pollution

## Abstract

Microplastics (MPs) and nanoplastics (NPs) have become pervasive contaminants in food, water, and air, leading to widespread human exposure, primarily through ingestion. Although MPs are increasingly detected in human tissues, including the placenta, blood, and brain, their long-term health implications are poorly understood. This review compiles emerging evidence on the systemic distribution and biological effects of ingested MPs, particularly on neurological risks. MPs can disrupt gut microbiota, breach intestinal and blood–brain barriers, and accumulate in neural tissues. Mechanistic studies reveal that MPs induce oxidative stress, neuroinflammation, protein aggregation, and neurotransmitter alterations, which may contribute to the development of cognitive dysfunction and neurodegenerative disease pathways. Recent work using brain organoids, single-cell and multi-omics technologies provides deeper mechanistic insights, linking MP/NP exposure to mitochondrial injury, inflammatory signaling, and impaired protein homeostasis. We also identify important gaps in exposure assessment, NPs detection, and epidemiological evidence. Human studies remain scarce but initial reports associating elevated MP/NP burdens in brain tissue with dementia highlight the urgency of this research. To address these gaps, we suggest critical next steps in the research agenda, integrating omics technologies, real-world exposure models, and human-relevant *in vitro* systems. As MP contamination grows, it is critical to understand its neurotoxic potential for informing public health policy and protecting vulnerable populations.

## Introduction

Microplastics (MPs), defined as plastic particles <5 mm (with nanoplastics (NPs) generally <1 μm) (1), arise either from the fragmentation of larger plastics (secondary MPs) or are manufactured as small particles (primary MPs, e.g., microbeads, resin pellets) (2). These particles are now pervasive across ecosystems, detected in marine and freshwater environments, soil, and air (3–5). Primary sources include single-use plastics, synthetic fibers, personal care products, and tire wear particles (4, 6, 7). Human exposure occurs through ingestion of contaminated food and water, inhalation and dermal contact (8–10).

MPs are ingested through diverse dietary items. They have been detected in seafood (particularly shellfish consumed whole), sea salt, tap and bottled water, and even fruits and vegetables (11–15). Cox et al. (16) estimated annual human ingestion at tens to hundreds of thousands of particles, equating to several grams per week. A 2019 study by Schwabl et al., found MPs in all analyzed stool samples, with a median of 20 particles per 10 g of feces (17), confirming routine dietary exposure. MPs have also been identified in human tissues such as the placenta and breast milk (18, 19) raising significant concerns about potential health impacts on infants and children. This review focuses on how ingested MPs enter and distribute in the body, their general health effects (on gastrointestinal, cardiovascular, immune, and reproductive systems), and, most critically, their emerging neurological implications.

To identify relevant literature, we conducted a structured search of PubMed, Web of Science, and Scopus for publications from 2015 through January 2025. Search terms included combinations of “microplastics,” “nanoplastics,” “neurotoxicity,” “ingestion,” “blood–brain barrier,” and “gut–brain axis.” We included peer-reviewed primary research and review articles that addressed exposure, toxicokinetics, systemic or neurological health effects, and human biomonitoring. Studies not published in English or lacking relevance to human or mammalian systems were excluded. References summarized in Tables 1–4 as well as those discussed in the main text, were identified through this process.

**Table 1:** Summary of key neurological findings from experiments across various animal models.

Animal model group	Plastic types and sizes^*^	Exposure methods	Key neurological findings	Major behavioral effects	References
Zebrafish Models (*Danio rerio*)	PS: 0.1–20 μm; PS-NPs: 1–10 μm; PS (~2 mg/L) ± Cu^2+^ (25 ug/L)	Water exposure: 5–30 days	Blood–brain barrier penetration; transcriptional changes in brain; elevated brain apoptosis; neurodevelopmental disruption; neuroinflammation; oxidative stress in brain tissue; altered dopaminergic signaling	Hyperactive swimming; altered predator avoidance; reduced locomotor activity; abnormal swimming patterns; social behavior alterations; cognitive impairment	(84–92)
Laboratory Rodents (Mice and Rats)	PS-NPs: 20–500 nm; PS-MPs: 5–20 μm; PE-MPs: 1–50 μm	Oral gavage (28–90 days); IV injection (24-72 h); intranasal (7–28 days)	BBB penetration; neuroinflammation via microglial activation; cerebral thrombosis; vascular obstruction; neuronal damage; altered neurotransmitter levels; astrocyte reactivity	Memory impairment; anxiety-like behavior; neurobehavioral abnormalities; motor dysfunction; altered exploratory behavior; olfactory dysfunction; learning/memory deficits	(70, 76, 93–97)
Fish Species (Goldfish, Carp, Medaka, Sea Bass, Tilapia, Trout)	PS-MPs: 1–500 μm; PE-MPs: 10–1,000 μm; Mixed MPs: 1–100 μm	Water/dietary exposure: 7–60 days	Brain accumulation; neuroinflammation; BBB dysfunction; lipid peroxidation; altered brain gene expression; neurodevelopmental toxicity; altered neurotransmitter metabolism	Altered swimming behavior; reduced feeding activity; behavioral alterations; stress responses; altered larval behavior; reduced survival; altered predator response	(98–105)
Invertebrate Models (*C. elegans*, *Drosophila*, *Artemia*)	PS-NPs: 20–200 nm; PS-MPs: 0.1–1 μm	Culture medium/food: 24 h-14 days	Neuronal dysfunction; altered neurotransmission; cholinergic system disruption; neurodegeneration; altered brain morphology; acute neurotoxicity; neuronal cell death	Reduced chemotaxis; altered feeding behavior; reduced climbing ability; altered circadian rhythms; reduced swimming activity; paralysis	(106–109)
Developmental Models (Pregnant mice, offspring)	PS-NPs: 100–500 nm	Oral gavage during gestation	Maternal-fetal brain transfer; placental transfer; developmental neurotoxicity; developmental disruption	Offspring behavioral abnormalities	(110, 111)

* PS, Polystyrene; PE, Polyethylene; MP, Microplastic; NP, Nanoplastic.

**Table 2:** Summary of human studies on microplastics/nanoplastics (MNPs) in the human brain.

Study type	MP/NP type*	Findings^*^	Population or sample	Citation
Autopsy Case Series—(first report of MPs in human brain tissue)	Microplastics (~5.5–26 μm particles; fibers ~21 μm). Identified polymers: mainly polypropylene (~44%), plus other synthetic fibers/fragments	Detected MPs in olfactory bulb of 8/15 cadavers, indicating that inhaled particles can reach the brain via the olfactory nerve pathway	Human brain (olfactory bulb)	(42)
Observational Clinical Study –(Blood–Brain-Barrier impairment study)	MP/NPs (PS, PE, PP, PVC)	All patients’ CSF contained some MPs (PP, PE, PS, PVC); patients with CNS infection (leaky BBB) had significantly higher CSF levels of PP and PE; Demonstrates that BBB damage allows greater MNP entry into the central nervous system	Cerebrospinal fluid from 28 hospital patients (14 with severe CNS infection and 14 without) in China;	(75)
Autopsy Cross-Sectional Study (Brain vs. other organs over time)	MPs and NPs (∼1 nm–500 μm). The predominant polymer was PE (≈75% of brain MNPs). Many particles were nanoscale “shard-like” plastic fragments	Higher MP burden in brain (~0.5% of brain tissue mass on average was plastic), than liver/kidney; accumulation over last decade; Brains of dementia patients contained ~6-fold higher microplastic concentrations than brains of non-dementia patients.	Human brains from 52 decedents in New Mexico, plus 27 archival brain samples	(43)
Case Report	Unspecified MPs	MPs detected in cerebral thrombi; speculated role in stroke	Cerebral blood clots	(70)
Tissue Biomonitoring	Plastic particles ≥700 nm	MPs detected in human blood; suggest systemic circulation	Whole blood from healthy adults	(34)
Theoretical/Review-Based	MPs with neurotoxic potential	Linked to Alzheimer’s, Parkinson’s via proposed mechanisms	Synthesized from animal/human data	(1)
Theoretical/Review-Based	MPs with neurotoxic potential	Proposed chronic microplastic exposure as a novel risk factor for dementia; mechanisms include oxidative stress, neuroinflammation, and amyloid aggregation	Synthesized from emerging human and animal evidence	(69)
Observational Clinical Study (CSF in Alzheimer’s disease vs. controls)	Micron-scale microplastics in CSF (identified polymers: PP, PVC, PE, PS); Frequency of bottled-water drinking correlated with higher CSF MP load.	Four polymer types of MPs were detected in the CSF of all subjects; AD patients had significantly higher CSF levels of PE and PVC than controls. In AD patients, higher CSF PE levels were linked to lower CSF Aβ42 (greater amyloid pathology), lower cognitive scores (MMSE) and faster cognitive decline.	Cerebrospinal fluid from 32 older adults in China: 17 amyloid-positive AD patients vs. 15 controls (baseline comparison), plus 11 additional AD patients in a validation cohort. All AD-diagnosed individuals were followed for 1 year of cognitive assessment.	(71)

* PS, Polystyrene; PE, Polyethylene; PP, Polypropylene; PVC, Polyvinyl chloride.

**Table 3:** Integrated mechanisms of micro- and nanoplastic (MNP)-induced neurotoxicity.

Mechanism	Key processes/molecular pathways	Supporting evidence (Refs)	Neurological outcomes
Oxidative Stress and Mitochondrial Dysfunction	ROS overproduction; electron leak at ETC, Complex I/III; NOX activation; redox-active additives catalyzing Fenton-like reactions; impaired Nrf2/Keap1 antioxidant signaling; lipid peroxidation (↑MDA/4-HNE); ferroptosis/cuproptosis features	(1, 32, 67, 76)	Memory deficits, neuronal death, cognitive impairment
Microglial Activation and Neuroinflammation	Microglial uptake of NPs → M1-like pro-inflammatory phenotype; cytokine release (IL-1β, TNF-α, IL-6); NOX2-driven ROS; impaired autophagy (↓LC3 flux, ↑p62) reducing clearance of aggregates	(32, 67, 72–74)	Chronic neuroinflammation, synaptic dysfunction, accelerated AD/PD pathology
Blood–Brain Barrier (BBB) Disruption	Tight junction protein loss (claudin-5, occludin, ZO-1); endothelial stress; cytokine-driven permeability; caveolin-1–mediated transcytosis	(33, 67, 75)	Increased brain permeability and microplastic/nanoplastic accumulation
Protein Aggregation and Misfolding	Particle corona promotes nucleation of α-synuclein, amyloid-β, TDP-43; lysosomal dysfunction (cathepsin leakage, impaired clearance); abnormal phase separation	(72, 77, 78)	AD: amyloid plaques; PD: Lewy bodies; Amyotrophic lateral sclerosis (ALS): TDP-43 inclusions
Neurotransmitter Dysregulation	Inhibition of AChE → ↑acetylcholine; altered dopamine/glutamate/GABA turnover; oxidative damage to monoamine oxidase (MAO), dopamine transporter (DAT), vesicular monoamine transporter (VMAT); ERK/MAPK signaling disruptions	(67, 76)	Cognitive decline, anxiety-like behaviors, locomotor changes
Gut–Brain Axis	Dysbiosis (↓short-chain fatty acid (SCFA) producers, ↑pro-inflammatory strains); ↓butyrate; gut barrier failure → endotoxemia (↑LPS); systemic cytokines; altered microbial amino acid metabolism (including possible effects on tryptophan pathways), bile acid shifts	(20, 44–48, 73)	Indirect neuroinflammation, impaired myelination, cognitive/behavioral dysfunction
Vascular and Clearance Pathways	Endothelial activation; platelet aggregation; cerebral thrombi formation; impaired glymphatic/lymphatic clearance of MPs	(33, 70, 75)	Stroke risk; chronic brain retention; neurodegeneration acceleration

ETC, electron transport chain; NOX, NADPH oxidase; Nrf2, nuclear factor erythroid 2–related factor 2; Keap1, Kelch-like ECH-associated protein 1; MDA, malondialdehyde; 4-HNE, 4-hydroxynonenal; LC3, microtubule-associated protein 1A/1B-light chain 3; p62, sequestosome-1; BBB, blood–brain barrier; AD, Alzheimer’s disease; PD, Parkinson’s disease; ALS, amyotrophic lateral sclerosis; AChE, acetylcholinesterase; GABA, gamma-aminobutyric acid; MAO, monoamine oxidase; DAT, dopamine transporter; VMAT, vesicular monoamine transporter; ERK/MAPK, extracellular signal-regulated kinase/mitogen-activated protein kinase; SCFA, short-chain fatty acid; LPS, lipopolysaccharide.

## Mechanisms of microplastic ingestion and absorption

### Sources of ingestion

Humans continuously ingest micro- and nanoplastics (MNPs) through food and beverages. Both tap and bottled water contain MPs, with plastic bottles showing particularly high loads (1). Heating liquids in plastic teabags or baby bottles can also release large quantities of MPs (20). Seafood, especially species consumed whole, are known vectors due to marine contamination (11, 21). Other foods, including salt, sugar, honey, beer, and produce, may contain MPs due to environmental or processing contamination (1). Additionally, indoor dust and synthetic fibers shed from textiles contribute to unintentional ingestion (22). Fang et al. (23) estimated that atmospheric deposition alone can contribute up to 1 million MPs per year to the human diet.

### Fate post-ingestion

Most MPs are excreted via feces; infants show significantly higher levels than adults (20). However, smaller particles, especially NPs, can cross the intestinal barrier (24–27). MPs < 150 μm may penetrate the gut lining, particularly via M-cells in Peyer’s patches and mucosal immune tissues (28, 29). While larger particles remain in the GI tract, smaller ones may enter circulation, depending on their size, charge, and surface chemistry. Continuous dietary exposure ensures a steady internal presence of MPs, underscoring the need to understand their bioavailability and health implications.

### Toxicokinetics of microplastics in the human body

Once ingested, MPs’ absorption and distribution are primarily governed by particle size and physicochemical properties (30, 31). Larger particles (>150 μm) are typically confined to the gastrointestinal tract and excreted, acting locally within the gut. In contrast, smaller MPs (<150 μm), especially NPs (<1 μm), can cross the intestinal barrier to some extent (31). Toxicological data estimate that ≤0.3% of small MPs may be absorbed, while NPs may achieve higher uptake, potentially several percent (31). Experimental studies confirm that polystyrene NPs between 20 and 100 nm can penetrate the intestinal lining and enter the bloodstream in rodents (32), likely through endocytosis or paracellular transport (33).

Once in the systemic circulation, MPs can travel to various organs. A landmark biomonitoring study detected particles ≥700 nm in human whole blood, including polyethylene and polyethylene terephthalate (PET), in 77% of donors (34). This confirms the systemic bioavailability of MPs in humans. Subsequent studies have found MPs in human lungs (35, 36), liver (37, 38), spleen (37), kidney (39), and placenta (40, 41). Notably, MPs were detected on both maternal and fetal sides of the placenta, demonstrating their ability to cross placental barriers.

Of critical concern is the brain. Animal studies have shown that NPs can cross the blood–brain barrier (BBB). For example, mice fed 30–50 nm polystyrene NPs showed brain accumulation and cognitive impairment (32). Two main routes are proposed: (1) via the bloodstream, where particles may breach the BBB by forming a protein corona or exploiting endothelial pathways (33), and (2) via the olfactory nerve, where inhaled particles migrate directly from the nasal cavity to the olfactory bulb (42). One autopsy case series detected polypropylene fragments in the olfactory bulbs of 8 of 15 human cadavers (42), suggesting direct nose-to-brain translocation.

Once in tissues, MPs may persist due to limited clearance. A 2023 autopsy study found higher concentrations of MPs in brain tissue than in the liver or kidney of the same individuals (43). Many particles were nanoscale, shard-like fragments consistent with environmental degradation products. Alarmingly, the total plastic burden in brains appeared to increase over the past decade (43). While clearance may occur via immune cells or the glymphatic system, recent findings suggest NPs may impair glymphatic clearance mechanisms (1).

Thus, the neurotoxic potential of MPs is strongly influenced by their physicochemical properties, particularly particle size and shape. Smaller NPs are more likely to cross biological barriers such as the intestinal epithelium and blood–brain barrier, while shape characteristics (e.g., rod-like or spiked forms) may enhance tissue penetration and cellular interactions. Table 4 summarizes how these properties affect brain accumulation and neurotoxicity based on current evidence.

**Table 4:** Influence of microplastic physicochemical properties on neurotoxicity and brain penetration.

Particle property	Typical size range	Key mechanisms involved	Neurotoxic outcomes	Evidence/citation
Smaller Size (Nano)	<1 μm	Enhanced translocation across intestinal and blood–brain barriers via endocytosis and paracellular transport	Greater brain accumulation; memory impairment; oxidative stress	(1, 32, 33)
Larger Size (Micro)	1–5,000 μm	Limited absorption; mostly retained in GI tract; local gut effects	Reduced neurotoxicity; gut dysbiosis with indirect brain effects	(30, 31)
Rod Shape	Variable	Higher surface area and binding potential; stronger cellular interaction	Increased brain uptake; sustained inflammation	(1)
Spherical Shape	Variable	Symmetric geometry; less membrane disruption	Lower uptake and accumulation in brain tissues	(1)
Sharp/Spiked Shape	Variable	Facilitated membrane piercing and internalization	Cellular damage, oxidative injury, possibly stronger neurotoxicity	(1, 26)
Chemical Composition	Varies by polymer	Different affinities for protein corona formation; affects immune recognition	Varied inflammatory responses and toxicity profiles	(33, 66)
Surface Charge and Chemistry	Variable	Influences interaction with cell membranes, protein corona, and biodistribution	Cationic surfaces associated with stronger toxicity	(33, 78)

Taken together, MPs show minimal absorption when large, but measurable systemic uptake when small, with the ability to cross biological barriers—including the placenta and BBB—and accumulate particularly in the brain. This underscores their potential for chronic internal exposure and associated neurological risks.

## General health effects of microplastic exposure

Although human data remains limited, growing evidence suggests that ingested MPs may pose risks to gastrointestinal, immune, cardiovascular, and reproductive health.

### Gastrointestinal (GI) tract

The GI tract is the primary site of contact with ingested MPs and is particularly vulnerable (44–46). Physical interactions between MPs and intestinal linings can cause irritation, inflammation, and even microlesions (45–47). Polystyrene MPs have been shown to disrupt intestinal integrity in animals and induce inflammatory responses (36, 47). A significant concern is the impact on the gut microbiome: MPs can lead to dysbiosis, shifting microbial balance toward pro-inflammatory organisms (44–48). These shifts are observed across species, from fish to rodents to humans, and beyond disrupting the microbiome, MPs also increase gut permeability, commonly referred to as ‘leaky gut. The weakening of tight junctions between intestinal cells (44–46) allows microbes and particles to translocate into circulation, potentially triggering systemic inflammation. These changes are associated with chronic disorders like inflammatory bowel disease and metabolic syndrome (45–47). Human data are still emerging, but the GI tract remains a critical site of concern for MP exposure with potential consequences extending along the gut-liver and gut-brain axes.

### Immune and inflammatory responses

MPs can elicit immune activation as foreign particles, especially when they cross mucosal barriers (49). Human immune cells internalize MPs *in vitro*. The result is the release of pro-inflammatory cytokines and Reactive Oxygen Species (ROS), a typical cellular response to MPs (49, 50). Persistent exposure may cause low-grade systemic inflammation. Chronic immune activation raises concern for links to autoimmune conditions, although direct evidence remains limited (51, 52).

Additionally, MPs can act as carriers for bacteria and toxins. Environmental MPs have been shown to adsorb pathogens and microbial metabolites, which may exacerbate immune responses (53, 54). They also bind heavy metals like lead and cadmium, but the health risks of such co-exposures remain underexplored (55, 56). Overall, MP-induced oxidative stress and immune activation likely underlie many health disturbances. These immune responses may not only affect peripheral systems but may also influence brain health. Chronic inflammation and cytokine signaling can disrupt the blood–brain barrier and contribute to neuroinflammation and neurodegenerative risks.

### Cardiovascular and metabolic effects

Though research is still in the early stages, there is growing concern about cardiovascular toxicity. MPs entering the bloodstream may damage the vascular endothelium and promote inflammation, a driver of atherosclerosis (57–59). MNPs have been detected in human atherosclerotic plaques, and higher burdens correlate with myocardial infarction, stroke, and mortality (58, 60). MPs also transport endocrine-disrupting additives (e.g., bisphenol A, phthalates) that are linked to obesity, insulin resistance, and cardiovascular disease (61). Disruption of gut microbiota by MPs may further exacerbate these effects through metabolic inflammation (61). Notably, MPs have been found in the cardiac tissues of patients undergoing surgery, though their pathological significance remains uncertain (59). Collectively, cardiovascular and metabolic disturbances provide plausible indirect routes to neurological harm via vascular injury, impaired cerebral perfusion, and systemic inflammation.

### Reproductive health

MPs have been detected in reproductive tissues, raising concerns about their impact on fertility and fetal development. The presence of MPs in the human placenta (“plasticenta”) suggests possible interference with placental function (62). Though the pregnancies in those studies were clinically normal, MPs may cause localized inflammation or oxidative stress that impairs nutrient exchange. Recent studies also found MPs in testicular tissue, correlating with reduced sperm quality (63). Endocrine-disrupting additives (e.g., phthalates, bisphenols) could further disrupt spermatogenesis and hormone signaling. Animal studies corroborate these risks: female or maternal exposure reduces fertility and offspring size (64). Maternal MP exposure has also been linked to lower birth weights and metabolic disturbances in offspring. While human data remain limited, reproductive and developmental effects are relevant to neurodevelopmental vulnerability, particularly via placental inflammation, endocrine disruption, and early-life metabolic programming.

### Other health considerations

Respiratory exposure to airborne MPs may contribute to lung inflammation or fibrosis (65). There is also concern about carcinogenesis. MPs can carry carcinogenic compounds like polycyclic aromatic hydrocarbons (PAHs), and chronic inflammation from particle exposure is a recognized risk factor for cancer (65, 66). However, human evidence for MP-induced carcinogenicity remains inconclusive. Most insights currently derive from *in vitro* systems or high-dose animal studies. Although MPs clearly have biological activity, their long-term effects in humans, especially at real-world exposure levels, require further investigation (66).

In summary, MP ingestion has been associated with multi-system inflammation and dysfunction. These gastrointestinal, immune, cardiovascular, and reproductive perturbations create conditions like systemic inflammation, endothelial/vascular injury, endocrine disruption, and microbiome-mediated signaling that potentially heighten vulnerability of the nervous system. We next examine mechanistic links to neurotoxicity.

## Neurological effects of microplastic exposure

Emerging evidence from experimental studies suggests that exposure to MNPs can lead to neurological impairments, including cognitive and behavioral dysfunctions. There are ethical and practical barriers to direct human studies, but animal models provide compelling insights. Numerous animal studies have provided mechanistic insights into how MNPs impair brain function. These studies span diverse species and exposure regimens, consistently reporting neurobehavioral changes, oxidative stress, and protein aggregation. Table 1 provides a summary of key experimental findings across various animal models.

In rodent models, oral exposure to polystyrene NPs (10–20 mg/kg/day) over several weeks has resulted in significant memory and learning deficits without affecting general health or motor function (32). These findings suggest subtle but specific neurobehavioral toxicity. Similarly, aquatic models such as zebrafish and nematodes have exhibited behavioral abnormalities, ranging from reduced exploration and impaired prey capture to locomotor disruption and convulsive activity at high MP concentrations (67). Collectively, these studies indicate that MPs may impair core neurological functions.

Human epidemiological data directly linking MP exposure to neurological outcomes are still lacking, but recent autopsy and case reports raise important concerns. Microplastics have been found in human brain tissue, including in individuals with dementia (43, 68). A recent autopsy study by Nihart et al. (43) reported that microplastic concentrations were significantly higher in human brain tissue compared to liver or kidney, and that dementia patients had markedly higher brain plastic burdens than non-dementia patients. Similarly, Gecegelen et al. (69) proposed chronic microplastic exposure as a novel risk factor for dementia. These findings provide compelling human evidence linking chronic microplastic accumulation to neurodegenerative risk. Although causality cannot be inferred from most cross-sectional findings, the accumulation of MPs in brain regions like the cortex and olfactory bulb (42) certainly raises the possibility of neurotoxic effects. Additionally, MPs have been detected in cerebral thrombi, prompting speculation that they may contribute to stroke risk by inducing microvascular obstruction (70). Detection of these particles in the human brain/CNS, although preliminary, raises important questions about chronic exposure and neurotoxicity. As summarized in Table 2, multiple recent human studies, including cerebrospinal fluid analyses (71) and brain autopsy series (43) provide direct clinical evidence of microplastics in the central nervous system. In their study He et al. reported microplastics in CSF along the AD continuum and linked higher CSF polyethylene and PVC to worse cognitive trajectories, reinforcing the clinical relevance of CSF plastic burden. These studies not only underscore the clinical relevance of microplastic neurotoxicity but also strengthen the rationale for investigating links with dementia and other neurodegenerative outcomes.

A particularly provocative area of investigation is the potential link between chronic MP exposure and neurodegenerative diseases. Preclinical studies suggest MPs may accelerate pathological processes underlying Alzheimer’s Disease (AD), Parkinson’s Disease (PD), and Multiple Sclerosis (MS). For instance, when in sufficient quantities, polystyrene NPs have been shown to promote alpha-synuclein aggregation, a hallmark of PD. (72) Other studies report that MPs facilitate amyloid-β aggregation *in vitro*, enhancing neurotoxicity in AD models (1). There is also evidence from fetal rat studies that MPs disrupt myelin formation, which could have relevance for MS. (1) While these disease-focused studies remain preclinical, they raise important hypotheses about MPs as environmental risk factors for neurodegeneration.

Cumulative evidence from animal studies suggests that MPs can impair memory, learning, and behavior and may promote the aggregation of neurotoxic proteins. These outcomes mirror features of neurodevelopmental and neurodegenerative disorders. Although most findings are from high-dose animal models, they raise critical questions about whether chronic, low-level human exposures could cause similar, albeit subtler, effects (67). Addressing this gap is essential, especially as MP contamination becomes increasingly pervasive. Overall, experimental data strongly support the neurotoxicity potential of MPs and underscore the need for more research.

## Molecular mechanisms of microplastic-induced neurotoxicity

**Figure 1:**
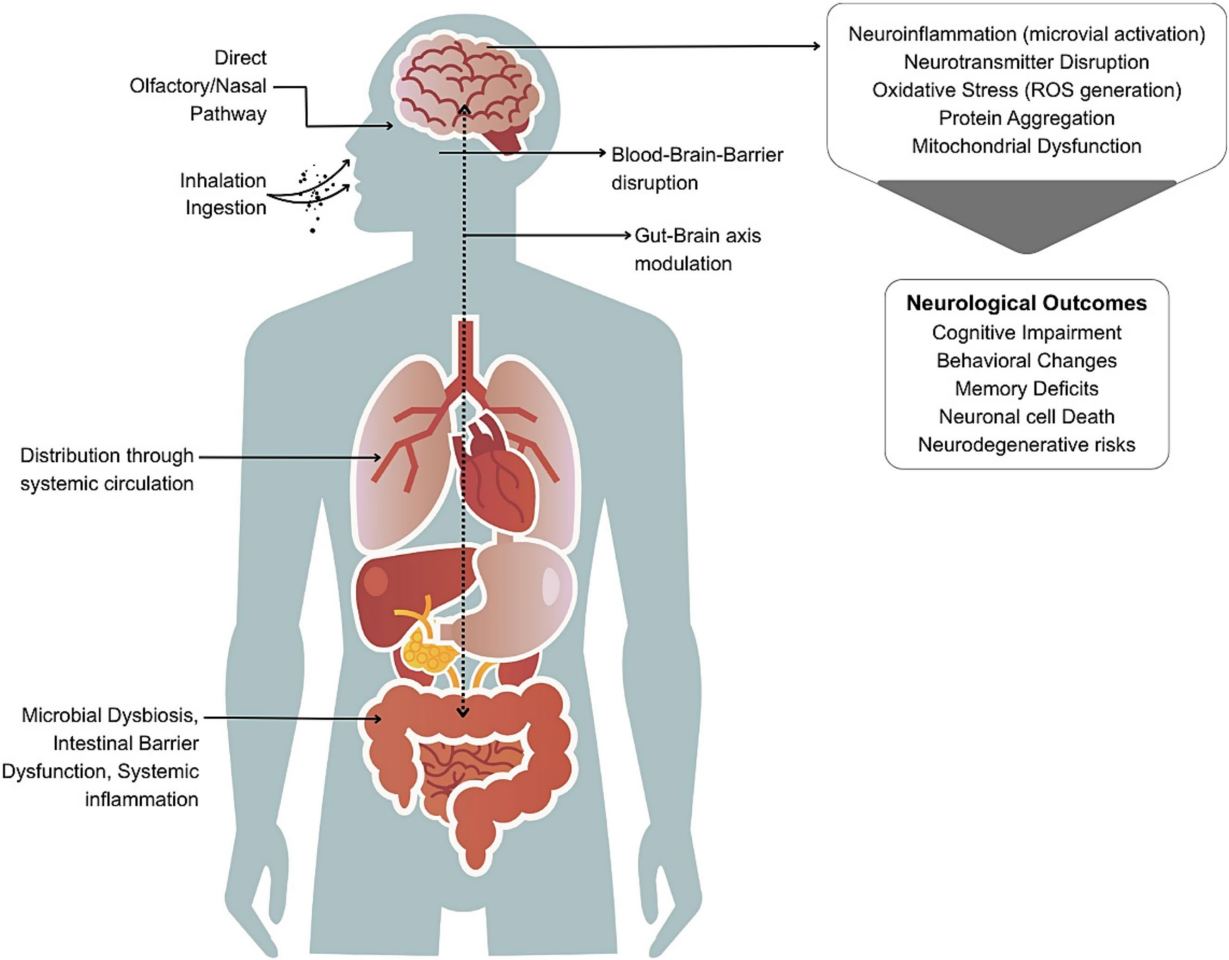
Pathways through which micro- and nanoplastics (MNPs) may cause neurological effects. MNPs from food, water, and air enter the body via ingestion or inhalation. Inhaled particles may bypass the blood–brain barrier (BBB) via the nasal/olfactory route. Ingested particles can disrupt gut microbiota and intestinal barriers, leading to systemic inflammation and translocation into circulation, ultimately affecting the brain through BBB disruption and gut-brain axis modulation. Once in the brain, MNPs may trigger neuroinflammation, oxidative stress, neurotransmitter imbalance, and protein aggregation contributing to cognitive, behavioral, and neurodegenerative outcomes.

The neurotoxicity of MNPs is based on interconnected biological processes. The mechanistic pathways can include oxidative stress, neuroinflammation, disruption of the blood–brain barrier (BBB), neurotransmitter dysregulation, protein aggregation, and/or modulation of the gut-brain axis (Figure 1). These mechanisms are discussed in detail below and an integrated summary of the key molecular mechanisms is presented in Table 3.

### Oxidative stress

Oxidative stress is a consistent and early response to MP exposure. Both animal models and *in vitro* studies show that MPs induce ROS generation in neuronal tissues (1, 67). Excessive ROS damages cellular biomolecules, leading to impaired neural function and cell death. Mechanistic studies indicate mitochondrial dysfunction as a central source of ROS, particularly electron leakage at complexes I and III of the electron transport chain, leading to loss of mitochondrial membrane potential and reduced ATP synthesis (1, 32). In rodents, oxidative injury in the hippocampus has been linked to memory deficits (32). ROS also activates signaling pathways like Nuclear Factor kappa-light-chain-enhancer of activated B cells (NF-κB), contributing to neuroinflammation and apoptosis. In addition, Nrf2/Keap1 antioxidant defenses appear downregulated in MNP-exposed neurons, suggesting impaired adaptive responses (67). Given its central role in neurodegenerative diseases MP-induced oxidative stress is considered a major mechanistic trigger of neural dysfunction (1).

### Neuroinflammation and microglial activation

MPs can provoke inflammatory responses once they enter the brain. Microglia, the brain’s resident immune cells, preferentially internalize NPs (32). They undergo morphological changes upon uptake and release pro-inflammatory cytokines and ROS, creating a neurotoxic environment. This is accompanied by activation of NADPH oxidase (NOX2), which amplifies oxidative and inflammatory signaling (67). Conditioned media from MP-exposed microglia has been shown to reduce neuronal firing activity, an effect reversible with anti-inflammatory inhibitors (32). Chronic microglial activation can damage neurostructures, driving disease progression. Additionally, impaired microglial autophagy, reflected in reduced LC3-II flux and p62 accumulation, further limits clearance of amyloid and α-synuclein aggregates, compounding proteostatic stress and thus exacerbating AD and PD pathology (72–74).

### Blood–brain barrier disruption

MPs can not only cross the BBB but also compromise its structural integrity. *In vitro* models reveal that polystyrene nanoparticles disrupt tight junction proteins in endothelial cells (33). Key targets include claudin-5, occludin, and ZO-1, whose downregulation increases paracellular permeability (33). Inflammatory cytokines released in response to MP exposure further degrade BBB tightness, potentially increasing brain exposure to other neurotoxicants (67). Sustained oxidative stress is another factor that weakens barrier function. Endothelial activation markers such as caveolin-1, VCAM-1, and ICAM-1 are also upregulated, suggesting active transcytosis and immune cell recruitment as additional routes of barrier compromise (67, 75). Thus, MPs may act both as direct neurotoxicants and facilitators of broader CNS vulnerability by impairing the brain’s primary defense.

### Neurotransmitter and synaptic effects

MNPs disrupt neurotransmitter systems. Studies have reported that inhibition of a Acetylcholinesterase (AChE) results in elevated acetylcholine levels at synapses, disrupting cholinergic signaling (67). This hypercholinergic state may disrupt normal long-term potentiation (LTP) and synaptic plasticity, processes essential for learning and memory. MPs also alter brain levels of dopamine, glutamate, and Gamma-Aminobutyric Acid (GABA) (1, 67). These neurochemical imbalances correspond with behavioral changes observed in exposed animals. In zebrafish and rodents, MP exposure has been associated with altered serotonin and dopamine signaling (67). Evidence also points to oxidative modification of dopamine transporter (DAT) and vesicular monoamine transporter (VMAT), which impair dopamine reuptake and storage (76). Enzymatic changes affecting neurotransmitter metabolism (e.g., monoamine oxidase inhibition) have also been reported. Together these suggest widespread disruption of synaptic communication.

### Protein aggregation and misfolding

Nanoplastics may serve as nucleation sites for the aggregation of neurodegeneration-related proteins. Experimental studies demonstrate that polystyrene NPs bind α-synuclein, accelerating its conversion to insoluble fibrils associated with Parkinson’s disease (77). Similarly, MPs promote amyloid-β aggregation, enhancing neurotoxicity in AD models (1). MPs also interfere with the normal degradation of proteins. Once internalized, they accumulate in lysosomes and impair their function, hindering the clearance of misfolded proteins (77). Lysosomal destabilization causes cathepsin leakage into the cytoplasm, further promoting neuronal apoptosis and inflammation (77). Promoting aggregation and inhibiting degradation contributes to toxic protein buildup, a hallmark of many neurodegenerative conditions. Additionally, NPs have been shown to induce TDP-43 aggregation, linked to amyotrophic lateral sclerosis (ALS) (78).

### Gut-brain axis and indirect effects

Ingested MPs may influence brain function indirectly via the gut-brain axis. MPs disturb the intestinal microbiome, reducing beneficial bacteria and increasing pro-inflammatory strains (20). Notably, depletion of butyrate-producing taxa reduces availability of short-chain fatty acids that are critical for maintaining gut barrier and microglial homeostasis (44–48). These microbiota shifts can affect brain health through altered production of microbial metabolites, e.g., short-chain fatty acids, amino acids and neurotransmitter precursors with potential downstream effects on neuroactive compounds (71) MPs also compromise gut barrier integrity. They promote systemic inflammation, a known contributor to neuroinflammatory and neurodegenerative processes. Behavioral and neural changes in MP-exposed rodents have been associated with these gut-level alterations (20, 73). Therefore, neurological consequences may result not only from MPs reaching the brain but also from cascading systemic effects originating in the gut.

### Integrated mechanisms

These mechanisms are not isolated. Oxidative stress can initiate microglial activation; neuroinflammation can impair BBB integrity, and disrupted autophagy can intensify protein aggregation. These synergistic interactions create positive feedback loops, for example, BBB disruption increases brain MNP accumulation, which further exacerbates oxidative and inflammatory stress. MPs can also alter membrane fluidity and intracellular signaling, which amplifies stress responses. Experimental studies using single-nucleus RNA sequencing in MP-exposed mice have revealed widespread transcriptional changes in neuronal pathways, particularly those regulating energy metabolism. This implicates mitochondrial dysfunction in MP-related neurotoxicity (79). Together, these findings indicate that MNPs are biologically active and capable of perturbing multiple molecular systems within the Central Nervous System (CNS). The cumulative effect of these disruptions may increase susceptibility to cognitive impairments, behavioral alterations, and progressive neurodegenerative diseases.

### Knowledge gaps

Despite rapid progress in understanding microplastic-induced neurotoxicity, several critical knowledge gaps remain. These are concerning human exposure levels, NPs detection, mechanistic specificity, and the effects of combined exposures and individual vulnerability.

### Human exposure levels and risk thresholds

We still lack precise data on typical brain exposures to MPs. MP intake has been quantified at tens of thousands of particles annually through food and water. They have been detected in blood and tissue (22, 34), but the internal dose required to cause neurological harm remains unclear. Moreover, the relationship between MPs’ physicochemical characteristics and their health impacts is poorly understood. Most toxicological studies use doses that exceed environmental exposure levels by orders of magnitude (67). Whether chronic, low-level exposures contribute to subtle neurofunctional changes has not been explored in humans. The absence of epidemiological studies linking MP exposure to neurodegenerative outcomes is a key barrier. This is partly due to the lack of validated exposure biomarkers. Future work should prioritize the development of sensitive, non-invasive biomarkers for MP burden.

### Detection of nanoplastics

A major technical challenge is the detection and characterization of NPs (<1 μm) in human tissues because most conventional analytical methods, such as micro-FTIR or Raman microscopy, have lower detection limits in the micrometer range (43). NPs, due to their small size and surface reactivity, are the most likely to cross biological barriers like the blood–brain barrier and accumulate in the brain (50, 51, 66). Their actual concentration in human tissues may be significantly underestimated. High-resolution pyrolysis gas chromatography mass spectrometry (py-GC/MS) or field-flow fractionation coupled with light scattering are needed to detect, quantify, and characterize NPs in biological matrices. Without such tools, risk assessments are likely to overlook the most neurotoxic fraction of plastic particles.

### Mechanistic specificity

While general mechanisms such as oxidative stress, neuroinflammation, and protein misfolding have been identified, our understanding of how specific MP characteristics drive these effects remains limited. Particle size, shape, charge, and polymer composition likely influence toxicity, but systematic comparisons are rare. For instance, whether spherical MPs are more neurotoxic than fibers or whether polystyrene elicits stronger microglial activation than polyethylene is not well established (67). Moreover, most mechanistic studies have been short-term. The potential for cumulative effects, such as protein aggregation, synaptic remodeling, or epigenetic changes, from chronic exposure has not been explored. Longitudinal studies and multi-omics approaches (transcriptomics, proteomics, metabolomics) could elucidate molecular pathways and identify markers of early neurotoxicity.

### Combined exposures and real-world conditions

Environmental MPs do not act in isolation. They often adsorb and transport other pollutants such as heavy metals, persistent organic pollutants (POPs), and microbial toxins (51, 66). Yet most laboratory studies use pristine, single-polymer spheres, which do not reflect the heterogeneous, weathered particles encountered in the environment (66). Surface oxidation, changes in hydrophobicity, and chemical loading can significantly alter toxicity profiles (80, 81). Studies comparing new vs. aged MPs and those incorporating adsorbed contaminants are urgently needed. For example, co-exposure models could test whether MPs carrying lead or per- and poly-fluoroalkyl substances (PFAS) have synergistic neurotoxic effects. Likewise, MPs may facilitate microbial translocation or endotoxin delivery across the intestinal or nasal mucosa, heightening immune responses (53, 54). Experimental designs must better mirror environmental conditions to ensure relevance to human health.

### Individual vulnerability and life stages

Susceptibility to MP neurotoxicity likely varies. Infants and children who ingest more MPs per body weight and have developing nervous systems may be particularly vulnerable (20). However, data on developmental neurotoxicity are virtually nonexistent. Do prenatal or early-life exposures affect long-term cognition? Maternal exposure studies suggest MPs can cross the placenta, but whether they impair fetal brain development remains unknown. Similarly, the role of MPs in accelerating age-related neurodegeneration is unexplored. Could the accumulation of NPs in aging brains worsen outcomes in AD or PD models? Genetic factors such as polymorphisms in oxidative stress pathways may also mediate susceptibility. These questions require targeted studies across life stages and in genetically diverse models.

### Thresholds, reversibility, and chronicity

It is unclear whether neurotoxicity from MPs exhibits a dose threshold or is reversible. Some rodent studies show effects at very low doses, while others require much higher exposure to elicit changes (67). This inconsistency suggests potential nonlinear or threshold-dependent effects. Longitudinal studies are needed to determine whether neural changes (e.g., inflammation or synaptic loss) resolve after exposure ends or persist, potentially leading to lasting dysfunction. Identifying whether damage accumulates over time or reaches a plateau will help refine risk assessments. Further, it is not known whether intermittent vs. continuous exposure has differential effects on brain accumulation and damage.

## Future directions

To advance the field of microplastic (MP) neurotoxicity and bridge critical knowledge gaps, a coordinated, interdisciplinary research agenda is essential. Below, we outline streamlined priorities that integrate epidemiology, exposure science, mechanistic toxicology, and public health policy.

### Advancing human exposure assessment and epidemiology

Robust epidemiological studies are urgently needed to evaluate the potential contribution of MP exposure to neurodevelopmental, neurobehavioral, and neurodegenerative outcomes. Currently, no population-level data link MP burden to diseases such as AD, PD, or cognitive decline, largely due to the lack of validated biomarkers of MP exposure. Research should focus on developing high-throughput, cost-effective methods to detect MNPs in biological matrices such as blood, urine, cerebrospinal fluid, and feces. These biomarkers must consider particle size, polymer type, surface properties, and adsorbed chemicals. Integrating such tools into existing cohorts (e.g., birth registries and aging studies) offers a scalable approach to human data generation.

### Improving nanoplastic detection technologies

The biological detection of nanoplastics remains technically challenging, particularly due to their small size and complex interactions with biological matrices. Spectroscopic techniques μFTIR and μRaman fail to detect the smallest, potentially most toxic particles. To move the field forward, efforts should focus on refining and standardizing these techniques for biological samples. Integrative strategies that combine imaging, spectrometry, and machine learning may enhance sensitivity and specificity. Establishing validated protocols and inter-laboratory benchmarks will be critical for generating reproducible, comparable data across studies.

### Mechanistic insights from organoid and *in vitro* systems

Advanced human-relevant *in vitro* systems, including neural organoids, microfluidic BBB models, and gut-brain-on-chip platforms enable detailed study of MP-induced neurotoxicity. Recent brain organoid studies show NPs reduce neural progenitors/neurons and perturb neurodevelopmental programs, underscoring translational relevance for human brain biology (82). These models support high-resolution investigations of particle size, polymer type, surface chemistry, and co-contaminant effects. Transcriptomic and proteomic profiling can identify early molecular changes preceding neurological damage. Studies using organoids from genetically susceptible donors (e.g., APOE4 for AD) can help uncover gene–environment interactions influencing vulnerability.

### Systems toxicology and multi-omics integration

A systems-level understanding of MP effects is needed. Multi-omics approaches; transcriptomics, metabolomics, epigenomics, and proteomics can help build integrated toxicity networks. For example, single-cell RNA-seq in MP-exposed brain tissue has highlighted disruptions in mitochondrial metabolism and synaptic signaling pathways (79). Emerging 2024 multi-omics work integrating brain transcriptomics with metabolomics similarly highlights synaptic and mitochondrial pathway disruption after MNP exposure, extending single-cell findings (83). Coupling omics data with functional assessments (e.g., behavior, electrophysiology) and applying machine learning can elucidate causal pathways and inform biomarker discovery.

### Transdisciplinary collaboration and stakeholder integration

Addressing MP neurotoxicity requires collaborative efforts across neuroscience, environmental health, materials science, microbiology, and computational biology. Equally important is engagement with policymakers, risk communication experts, and communities. Transdisciplinary centers and consortia can facilitate data sharing, method harmonization, and consensus-building on exposure thresholds. Including citizen science and open-access databases can increase transparency, trust, and relevance of findings.

### Enhancing real-world relevance of exposure models

Toxicological studies often use pristine MPs, which differ from environmentally aged particles present in food, air, and water. These aged MPs exhibit surface oxidation, biofilm accumulation, and chemical adsorption that alter biological interactions (66). Future models must simulate realistic exposure conditions, including mixed MPs, co-contaminants, and chronic low-dose regimens. Studies should also assess the bio-corona that forms *in vivo* and its role in MP uptake and immune interactions.

### Identifying vulnerable populations and windows of susceptibility

The developing brain is especially vulnerable to environmental insults. Prenatal and early-life exposure to MPs may disrupt neurodevelopment, as evidenced in animal studies showing impaired myelination and neuronal differentiation (1). Aging populations may also be at risk due to cumulative MP burden and comorbidities. Research should evaluate sex-based, genetic, and life-stage differences in MP absorption, distribution, and toxicity. Stratification by risk profiles will enhance the precision of epidemiological insights and interventions.

### Exploring combined effects with environmental co-stressors

MPs often act synergistically with other pollutants, enhancing the bioavailability and toxicity of co-adsorbed chemicals such as heavy metals, PFAS, or microbial toxins (51, 54, 66). Additionally, MPs may compromise host defenses, including the gut microbiome, immune system, and blood–brain barrier. Future research must adopt multi-stressor models that mirror real-world exposures and uncover interactive effects on neurological health.

### Bridging science and policy for risk reduction

Scientific findings must inform actionable regulations. Despite recognizing MP contamination, bodies like the World Health Organization (WHO) and the European Food Safety Authority (EFSA) have not yet issued enforceable health-based guidelines due to limited toxicological data. Research should help establish evidence-based exposure limits, prioritize high-risk plastic sources, and guide interventions (e.g., safer food contact materials, improved water filtration, waste reduction policies). Scientists must engage early with regulators to ensure timely translation of findings. Public outreach and educational campaigns can empower consumers to adopt exposure-reducing behaviors, especially among high-risk groups like pregnant women and children.

## Conclusion

Long-term health impacts are a pressing concern, particularly in the brain, because microplastics are inescapable. They are pervasive in the environment and have been detected in human tissues. Experimental studies provide compelling evidence of microplastic-induced neurotoxicity but direct evidence in humans remains limited. Addressing this problem will require research integrating human exposure assessment coupled with advanced *in vitro* and omics-based tools and real-world toxicological models. Moving beyond laboratory findings toward translational science that informs public health and regulatory action is essential. Ultimately, understanding and mitigating the neurological risks of microplastics is not only a scientific imperative but a public health priority.
